# Profiling Protein Citrullination in Extracellular Vesicles by Single‐Molecule Detection Using Direct Stochastic Optical Reconstruction Microscopy

**DOI:** 10.1002/jbio.202500483

**Published:** 2025-11-27

**Authors:** Sarah R. Needham, Benjamin M. Davis, Pinar Uysal‐Onganer, Daniel J. Rolfe, Mariya Hristova, Igor Kraev, Jameel M. Inal, Sigrun Lange

**Affiliations:** ^1^ Central Laser Facility, UKRI: Science & Technology Facilities Council, Rutherford Appleton Laboratory Oxfordshire UK; ^2^ Cancer Mechanisms and Biomarkers Research Group, School of Life Sciences, University of Westminster London UK; ^3^ Department of Neonatology EGA Institute for Women's Health, University College London London UK; ^4^ Electron Microscopy Suite, Faculty of Science, Technology, Engineering and Mathematics, Open University Milton Keynes UK; ^5^ Cell Communication in Disease Pathology, School of Human Sciences, London Metropolitan University London UK; ^6^ Pathobiology and Extracellular Vesicles Research Group, School of Life Sciences, University of Westminster London UK

**Keywords:** Bayesian models, citrullinated histone H3, citrullination/deimination, dSTORM, extracellular vesicles, liquid biopsy, single‐molecule detection

## Abstract

Extracellular vesicles (EVs) are critical in cellular communication and pathological biomarkers. Post‐translationally deiminated/citrullinated proteins are reported in EV cargoes by LC–MS/MS but it is unknown in which EV sub‐types they are exported, as part of EVs' intraluminal cargo or on the EV surface. Here, dSTORM super‐resolution microscopy is used to co‐localise total citrullinated proteins (pan‐Cit), and citrullinated histone H3 (CitH3) to EV subtypes of three cancer cell lines, captured by tetraspanin trio (TT) or phosphatidylserine (PS). Permeabilised and non‐permeabilised EVs are analysed with a Bayesian framework using beta‐distributed posteriors for binomial outcomes. Pan‐Cit and CitH3 labelling is confirmed in EVs as intraluminal cargo and on the EV surface, with higher levels detected in the permeabilized EVs. Pan‐Cit staining is higher in TT‐bound EVs, but CitH3 staining higher in PS‐bound EVs. This study expands the landscape of EV‐associated post‐translational modifications with translational potential for EV‐citrullinome based liquid biopsy tools.

## Graphical abstract

This study identifies post‐translationally citrullinated/deiminated protein cargoes co‐localised to EV subtypes, using super resolution microscopy and dSTORM imaging. The findings expand current understanding of post‐translational modifications in EV‐mediated cellular communication. The Bayesian framework presented here provides a new tool for modelling EV cargoes to EV subtypes, aiding the development of refined EV‐based liquid biopsy tools.

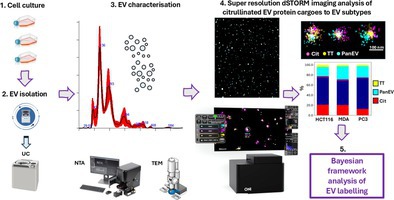

## 1 Introduction

Extracellular vesicles (EVs) are 30–1000 nm lipid bilayer structures, which are released from cells as a critical part of cellular communication, including via the transport of various cargoes such as non‐coding RNAs, proteins and post‐translationally modified proteins [1, 2, 3, 4]. EVs play important roles in pathobiological processes, including in cancer crosstalk via the export of pro‐cancerous cargoes that can contribute to cancer progression and metastasis [5, 6]. In cancer, the identification of increased EV release and modified EV cargoes has highlighted EVs and their specific cargo contents as valuable biomarkers [7, 8].

Protein citrullination/deimination is a post‐translational modification of arginine to citrulline, caused by peptidylarginine deiminases (PADs), a family of five calcium‐activated isozymes in humans, which differ in tissue distribution and target protein preferences [9, 10, 11]. Citrullination leads to structural and functional changes in target proteins, contributing to neo‐epitope formation, which can induce inflammatory responses related to many inflammatory and autoimmune diseases, as well as cancer [12, 13, 14, 15, 16, 17]. Furthermore, citrullination of histones can contribute to epigenetic changes, including via histone H3 citrullination which has been identified in various cancers, and CitH3 has been highlighted as a circulatory cancer biomarker [18, 19, 20]. CitH3 is also a marker of neutrophil extracellular trap formation (NETosis), which is linked to many inflammatory diseases, including cancer and cancer‐associated thrombosis [21, 22, 23].

Roles for protein citrullination have been identified by us and others in a range of cancers [24, 25, 26, 27, 28, 29]. Importantly, previous research from our group has identified roles for PADs and citrullination in EV biogenesis and modulation of EV cargoes in several cancer types [24, 25, 30, 31] and analysed post‐translationally deiminated/citrullinated protein cargoes in EVs by proteomic analysis. While these previous studies, using LC–MS/MS, revealed a range of citrullinated proteins in EV cargoes of various biofluids across taxa [32, 33, 34, 35, 36, 37, 38, 39, 40, 41, 42, 43], including CitH3, it has yet to be established which exact EV subtypes export the majority of citrullinated proteins. Also, it needs to be identified whether citrullinated proteins are transported by EVs as intraluminal EV cargo or if they form part of the EV corona. Furthermore, as CitH3 has been highlighted as a circulatory biomarker in cancer [18, 19, 20], it is of considerable interest to assess CitH3 export as part of EV cargo, including with respect to EV subtypes.

The use of dSTORM super‐resolution microscopy [44] allows for co‐localisation of EV‐specific markers and cargo proteins of interest, which is of significant importance for accurate biomarker development based on quantification of specific EV cargoes at the single molecular level [45, 46]. Therefore, this method was used in the current study to co‐localise total citrullinated proteins and CitH3 within EV subtypes, using pan‐EV staining and tetraspanin trio staining, and tetraspanin or phosphatidylserine (PS) capture, respectively. This approach allows for capture of tetraspanin‐positive and PS‐positive EVs, followed by co‐labelling with EV surface markers and specific protein cargo markers of interest.

To quantify uncertainty and compare EV labelling extent across experimental conditions, a Bayesian framework using beta‐distributed posteriors for binomial outcomes was adopted [47]. This approach is particularly well suited to problems involving count or event data, especially when sample sizes are small or when zero‐event cases occur. By treating observed counts as evidence updating a prior belief (modelled here with a uniform Beta(1,1) prior), posterior distributions are obtained over the underlying success probabilities. These posteriors enable direct probabilistic reasoning, such as estimating credible intervals or computing the probability that one condition outperforms another, without relying on asymptotic approximations or *p* values. This method provides both robustness and interpretability when comparing proportions between groups.

We hypothesise that citrullinated/deiminated proteins are exported via EVs both as intraluminal cargo, as well as part of the EV corona.

## 2 Experimental Section/Materials and Methods

### 2.1 EV Isolation

EVs were isolated from cell culture medium of two cancer cell lines: prostate cancer (PC3; ATCC CRL‐1435) and breast cancer (MDA‐MB‐231; ATCC HTB‐26) cells according to previously published methods [27, 30, 48], adhering to the MISEV2023 guidelines [2]. Both cell lines were obtained from the American Type Culture Collection (ATCC, USA) and maintained in RPMI‐1640 or Dulbecco's Modified Eagle Medium (DMEM) supplemented with 10% fetal bovine serum (FBS) and 1% penicillin–streptomycin, for the PC3 and MDA‐MB‐231 cell lines respectively. The cells were cultured at 37°C in a humidified incubator with 5% CO_2_. For EV isolation, cells were grown to 80% confluence in T75 flasks; the standard medium was then removed and replaced with serum‐free medium to avoid contamination with FBS‐derived EVs and returned to the cell incubator for 3 h. Following the 3 h incubation in serum‐free medium, the media were collected (10 mL per flask; 5 flasks per cell line) and spun at 4000 *g* for 20 min to remove any cells and aggregates. The supernatants were then ultracentrifuged at 100,000 *g* for 1 h at 4°C to collect the EV‐enriched pellets. The supernatants were discarded, the EV pellets were washed in 1 mL DPBS (filtered through a sterile 0.22 μM filter) and ultracentrifuged again at 100,000 *g* for 1 h at 4°C. The DPBS was discarded, the final EV pellets (derived from a total of 50 mL sample pool per cell line) were diluted in 100 μL sterile filtered DPBS each. The EVs were quantified by nanoparticle tracking analysis (NTA) to determine sample concentration, assessed for positive detection of two surface markers (CD63 and flotillin‐1) by western blotting and visualised by transmission electron microscopy (TEM) before further analysis by dSTORM imaging. EVs from the HCT116 cell line provided with the EV2 profiler kit (ONI) were used as standard EVs for all dSTORM analyses.

### 2.2 EV Characterisation by NTA, WB and TEM

EVs were quantified and size profiles generated by NTA using the NS300 Nanosight (Malvern Panalytical Ltd., UK) equipped with a sCMOS camera and a 488 nm diode laser. EV samples were applied to the system with an automatic syringe pump set at speed 50; camera levels were set at 12 for capture and at threshold level 5 for post‐analysis processing. Each EV sample was recorded for 4 × 60 s, and histograms representing mean and standard error were generated by averaging the four readings per sample using the NTA software (version 3.0., Malvern Panalytical Ltd.). The NTA analysis reports were used to quantify EV concentration (EVs mL^−1^), and to assess median and modal EV sizes per sample.

Western blotting was used to assess two EV surface markers, CD63 (ab216130, Abcam, UK) and Flotillin‐1 (ab41927, Abcam), as previously described [30, 48]. Protein separation was carried out using 4%–20% gradient TGX gels (BioRad UK) followed by Western blotting using a Trans‐Blot SD semi‐dry transfer cell (BioRad, UK). Membranes were blocked with 5% bovine serum albumin (BSA, Sigma, UK) in Tris‐buffered saline (TBS) containing 0.1% Tween20 (BioRad, UK; TBS‐T) for 1 h at room temperature (RT), followed by primary antibody incubation overnight at 4°C, washing in TBS‐T and incubation at RT for 1 h with HRP‐conjugated anti‐rabbit IgG (BioRad, diluted 1/3000 in TBS‐T). The membranes were washed in TBS‐T and visualised using enhanced chemiluminescence (ECL, Amersham, UK) with digital images acquired by the UVP BioDoc‐ITTM System (Thermo Fisher Scientific, Dartford, UK).

The EVs were imaged by transmission electron microscopy (TEM) following resuspension in 100 mM sodium cacodylate buffer (pH 7.4), applying 3–5 μL of the EV suspension to a glow‐discharged TEM grid with a carbon support film. The sample was partially air‐dried for approximately 10 min before the grid was placed onto a drop of fixative solution (2.5% glutaraldehyde (Agar Scientific Ltd., Stansted, UK) in 100 mM sodium cacodylate buffer, pH 7.4) for 1 min at room temperature. The grid was subsequently transferred across three drops of distilled water for washing, with excess water being removed using filter paper. Then the grid was placed onto a drop of staining solution of 2% aqueous Uranyl Acetate (Agar Scientific Ltd., Stansted, UK) for 1 min, and any excess stain was removed with filter paper before air drying. Transmission electron microscopy (TEM) imaging of the EVs was conducted using a JEOL JEM 1400 microscope (JEOL, Tokyo, Japan) operated at 80 kV, with magnifications ranging from 30 000× to 60 000×. Digital images were captured using a GATAN Rio16 digital camera (Ametek GB Limited, Leicester, UK).

### 2.3 Direct Stochastic Optical Reconstruction Microscopy (dSTORM) for Super‐Resolution Imaging of Citrullinated EV Cargo Proteins and EV Markers

Single‐molecule localisation‐based super‐resolution microscopy imaging of the cancer EVs (HCT116, PC3 and MDA‐MB‐231) was carried out to visualise EV surface markers and the EV cargo proteins of interest: pan‐citrullinated proteins and CitH3, which were labelled with fluorophorescently tagged antibodies. The antibodies for pan‐citrullination (F95, Merck, Feltham, UK) and citrullinated histone H3 (CitH3, Abcam, Cambridge, UK) were conjugated to AlexaFluor‐647 in‐house (STFC, RAL, Harwell, UK) to use for co‐labelling experiments on EV chips from ONI (Oxford, UK). EVs derived from colon cancer cells (HCT116) served as positive controls (ONI), while no EVs were added to one lane per chip as a negative control; and the EVs isolated from the PC3 and MDA‐MB‐231 cells were used under the same conditions and chip for each experimental setup. The ONI EV Profiler 2 kit and was used for EV labelling and the ONI Nanoimager system for visualisation of the EVs by dSTORM (ONI UK, Oxford, UK). Samples were prepared according to the manufacturer's instructions (ONI), following the manual sample preparation protocol (EV Profiler 2). Briefly, either tetraspanin trio (TT) or phosphatidylserine (PS) was used to capture EVs to the chip surface. Chips were prepared both for permeabilised EVs as well as for non‐permeabilised EVs (according to the manufacturer's instructions) to establish whether the cargoes were intraluminal or on the EV surface (EV corona). EV visualisation was carried out by labelling with tetraspanin trio (anti‐CD9 + CD63 + CD81 (561)) and a pan‐EV marker (488), with the cargo proteins of interest (pan‐citrullinated proteins or CitH3 labelled with Alexa‐647 fluorophore) and co‐localised to EV subtypes. Fresh dSTORM imaging buffer was applied to the chips which were immediately imaged using the Nanoimager S Mark II system with the AutoEV imaging and CODI (https://alto.codi.bio/) analysis protocol according to the manufacturer's specifications (ONI). Laser settings were as follows for imaging: 180 mW of 488 nM laser, 44 mW of 561 laser and 170 mW of 640 nM laser. Imaging was carried out at 40 ms/frame with 3000 frames for the 488 nm laser, and 1000 frames for the 561 and 640 nm lasers, in six fields of view for each chip per sample, to stay within the duration of the 90 min span of the dSTORM buffer being active. Each chip was imaged twice (with fresh dSTORM buffer applied for the second imaging), gathering information of a total of 12 fields of view per sample. Initial data reports were generated by AutoEV, reporting the proportion of labelled and co‐labelled EVs from each cell line for each condition.

### 2.4 Quantification of Labelled Extracellular Vesicles and Statistical Analysis

For quantification of labelled EVs, datasets (EV positivity) were recovered using the manufacturer's recommended Particle filters: diameter < 500 nm, label counts > 5 for EV characterisation markers (pan‐EV and TT), and > 2 for the protein marker of interest (panCit or CitH3, respectively). To account for experiment variability, all measurements were performed from 6 to 12 fields of view, in at least two independent replicates.

EV detection was assessed via the Kolmogorov–Smirnov Test: To ensure genuine signal over background, a one‐tailed, two‐sample Kolmogorov–Smirnov (KS) Test was performed, comparing the number of EVs detected in each field of view against its corresponding negative control distribution. A KS statistic *p* value < 0.01 (one‐tailed testing, for a substantial positive shift in the distribution of EV counts versus the negative control) was taken as confirmation of substantial EV population detection in that condition compared to negative controls. Failure to detect a significant population of EVs over negative controls precluded further analysis of this experimental condition.

#### 2.4.1 Uncertainty Estimation

To estimate particle count uncertainties, EV mean label counts from each chip were treated as alpha and beta terms of a Beta distribution. Specifically, as *α* represented counts in the presence of citrullination and β represented counts in its absence for each particle type, yielding a posterior with mean E[X] = *α*/(*α* + *β*) and variance Var[X] = αβ/(*α* + *β*)^2^(*α* + *β* + 1). A uniform prior was used in all cases, corresponding to Beta (*α* = 1, *β* = 1).

To explain differences between experimental conditions in terms of observed proportions a Bayesian approach was employed using beta‐distributed posteriors for binomial outcomes. Let *α*_1_ and *β*_1_ denote the successes and failures, respectively, in n_1_ trials under one condition, and *α*_2_, *β*_2_ and *n*_2_ denote the same under a second condition. Posterior distributions, p_1_(x) and p_2_(x), for the underlying success probabilities, x, were modelled as independent Beta distributions with parameters Beta(*α*_1_, *β*_1_) and Beta(*α*_2_, *β*_2_) respectively. Two key metrics were computed:

1. Posterior difference estimation: To estimate the distribution of the difference *p*_1_—*p*_2_ 200 000 Monte Carlo samples were drawn from each posterior. The mean and 95% credible interval (CI) of the resulting difference distribution for reporting were calculated.

2. Probability that one condition outperforms the other: The posterior probability that *p*_1_ > *p*_2_ was computed using the integral:

$$\begin{array}{c} P\left(p_{1}>p_{2}\right)=\iint_{x=0,y=0}^{x=1,y=x}p_{1}\left(x\right)p_{2}\left(y\right)\text{dxdy}=\int_{0}^{1}p_{1}\left(x\right){\mathrm{CDF}}_{2}\left(x\right)\mathrm{dx} \end{array}$$

where *CDF*_2_(x) is the cumulative distribution function (CDF) for p_2_(x). This integral was evaluated using numerical quadrature.

The approach provides both an interpretable estimate of effect size and a probabilistic statement about comparative performance, even under low‐data or zero event scenarios. These tests were used to compare effect sizes between groups or between groups and a null hypothesis of no citrullination detected (i.e., *α*_null_ = 0 and *β*_null_ = *β*_1_).

#### 2.4.2 EV Size Determination From dSTORM Localisations

EV diameters were computed from the Pan‐EV membrane‐label channel only. Single‐molecule localisations were drift corrected and clustered using CODI's EV‐specific spatial density and circularity thresholds. Clustering was determined by DBSCAN after which each cluster's two‐dimensional outline was estimated by a convex/alpha hull of the Pan‐EV localisations and the enclosed area (A) was converted to an equivalent circular diameter (d), by:

$$d=2\surd \left(A/\pi \right)$$

Localisation precision (xy) was 15–20 nm under these imaging conditions (*σ*_loc_), corresponding to an estimated systematic size bias of 8 to 14 for 50 nm diameter EVs, decreasing to 3.2 to 5.2 nm for 150 nm diameter EVs. This is based on the assumption,

$$d_{\text{observed}}=2\surd {R_{\text{actual}}}^{2}+{\sigma^{2}}_{\mathrm{loc}}$$

where *R*_actual_ is the actual EV radius and d_observed_ is the observed particle diameter.

In addition, for a roughly circular EV, an additional uncertainty from the random per‐EV spread from finite sampling will become substantial when the number of observations is low. By default, CODI defines an EV as any cluster containing ≥ 5 localisations (*N*). This threshold is appropriate for EV detection but may not be sufficient for accurate quantitative size estimation, as the footprint defined by five localisations is dominated by localisation precision rather than vesicle geometry. An obvious solution is to increase *N*; however, *N* is not a reliable proxy for geometric sampling quality in this application of dSTORM as the number of times each fluorophore can be measured varies, generating multiple localisations from the same physical emitter and so should not be used as independent estimators of EV positional uncertainty. As all samples were acquired and analysed under identical imaging and clustering conditions, any systematic inflation of apparent size is expected to affect all groups equally. Comparative trends between conditions remain meaningful, whereas absolute diameters should be interpreted cautiously.

#### 2.4.3 Particle Size Distribution Analysis

It was assessed whether two empirical EV populations differed in their overall particle size distributions using the two‐sample Kolmogorov–Smirnov (KS) test. This non‐parametric test evaluates the null hypothesis that the two samples are drawn from the same continuous distribution, without assuming any particular distributional form. If a significant difference was detected (e.g., *p* < 0.05), the analysis was proceeded to estimate the magnitude of the difference in medians between the two populations using a bootstrap procedure. Specifically, for two samples *a* and *b*, 10 000 bootstrap replicates were generated by resampling each group with replacement. For each replicate, the difference in medians was computed, and the 2.5th and 97.5th percentiles of the resulting distribution were taken as the 95% confidence interval for the median difference. This approach provides a robust, distribution‐free estimate of the effect size and its associated uncertainty, complementing the KS test's binary decision with an interpretable summary of magnitude.

All analyses were conducted using Python (3.9) with packages numpy (1.25.2), scipy (1.11.1) and pandas (1.5.3).

## 3 Results

### 3.1 Characterisation and Quantification of Extracellular Vesicles From PC3 and MDA‐MB‐231 Cells

**FIGURE 1:**
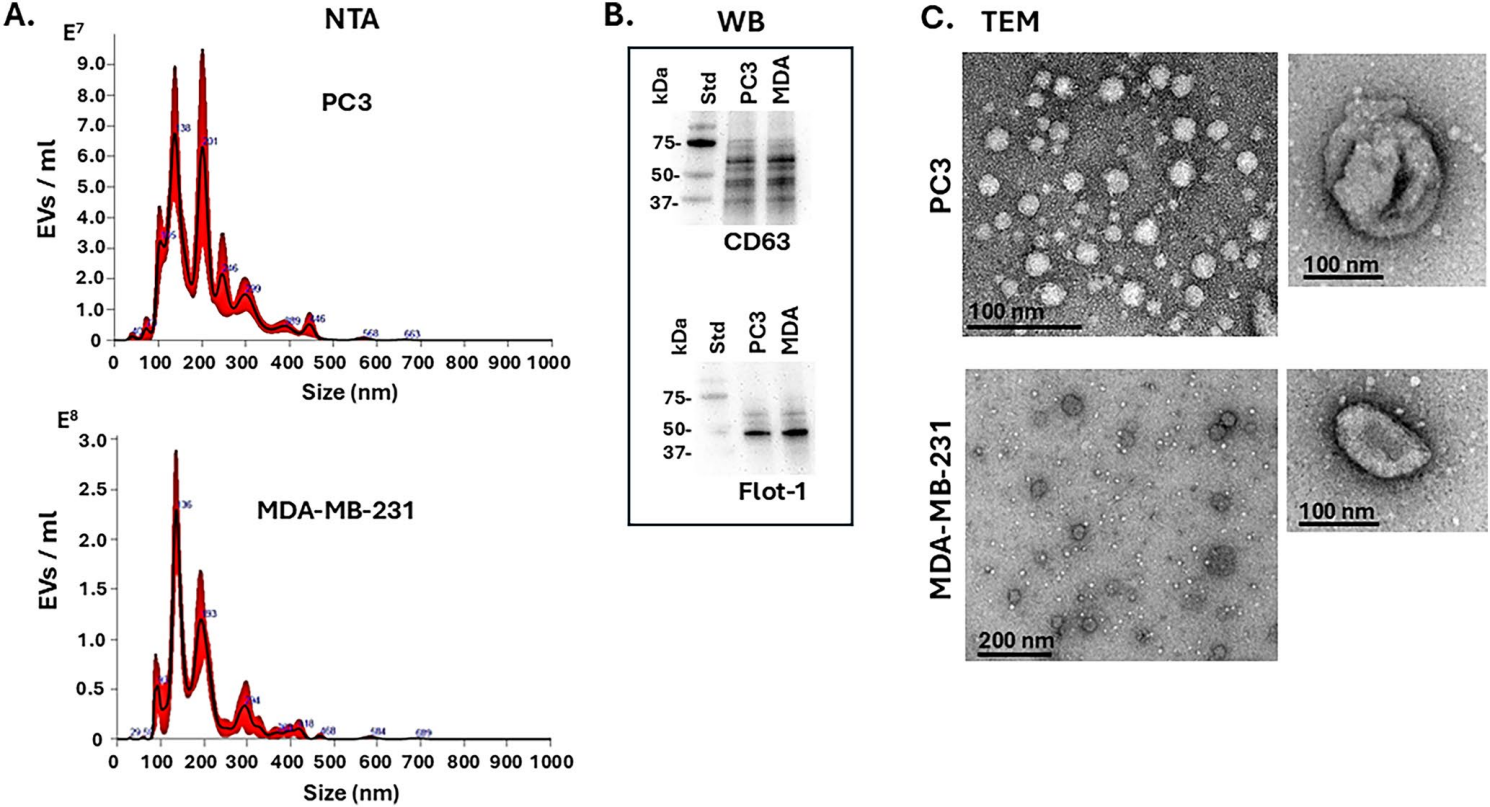
Characterisation of EVs isolated from PC3 and MDA‐MB‐231 cells. (A) Nanoparticle tracking analysis (NTA) showing EV size distribution profiles; (B) EV surface marker detection for CD63 and flotillin‐1 (Flot‐1) by western blotting (WB), indicating the size standard (Std) in kDa; (C) Representative transmission electron microscopy (TEM) images for EVs isolated from PC3 and MDA‐MB‐231 cell cultures; scale bars are included, representing 100 or 200 nm.

Characterisation of EVs isolated from PC3 and MDA‐MB‐231 cells was carried out using nanoparticle tracking analysis (NTA) which verified poly‐dispersed EV populations in the size range of 30–450 nm for both experimental cancer cell lines, with a modal size of 137 nm (SD 84.4 nm) for PC3 EVs and 136 nm (SD 80.5) for MDA‐MB‐231 EVs (Figure 1A). EV surface marker detection for CD63 and flotillin‐1 showed positive results for both EV preparations by western blotting analysis (Figure 1B). The EVs were furthermore imaged by transmission electron microscopy (TEM; Figure 1C). EV quantification confirmed that the isolated EV samples were in the range of 1 × 10^9^ to 1.5 × 10^10^ mL^−1^, which is the recommended EV concentration for application on the EV profiler kit (ONI, Oxford, UK).

### 3.2 Detection of Pan‐Citrullinated and Citrullinated Histone H3 Protein Cargoes in Cancer Cell EVs by Single Molecule Detection using dSTORM Imaging

The extent of EV labelling between groups was evaluated by a Bayesian framework using beta‐distributed posteriors for binomial outcomes. This allowed for accurate comparison of EVs labelled for pan‐citrullination (panCit), or citrullinated histone H3 (CitH3), comparing permeabilised EVs (detecting both intraluminal target proteins in EV cargo as well as target proteins on the EV surface), versus non‐permeabilised EVs (detecting the target proteins on the EV surface (EV corona)).

EVs were imaged by dSTORM for the detection of either panCit or CitH3, and co‐labelled with tetraspanin trio (TT) and pan‐EV marker. The analysis was carried out on EVs that had been captured on the ONI chips with either TT or PS capture and on EVs that had been permeabilised, compared to EVs that were non‐permeabilised.

#### 3.2.1 Distribution of Citrullinated Protein Cargoes in Cancer Cell EVs, Localised to EV Subpopulations and Permeabilised Versus Non Permeabilised Conditions, as well as TT or PS Capture

**FIGURE 2:**
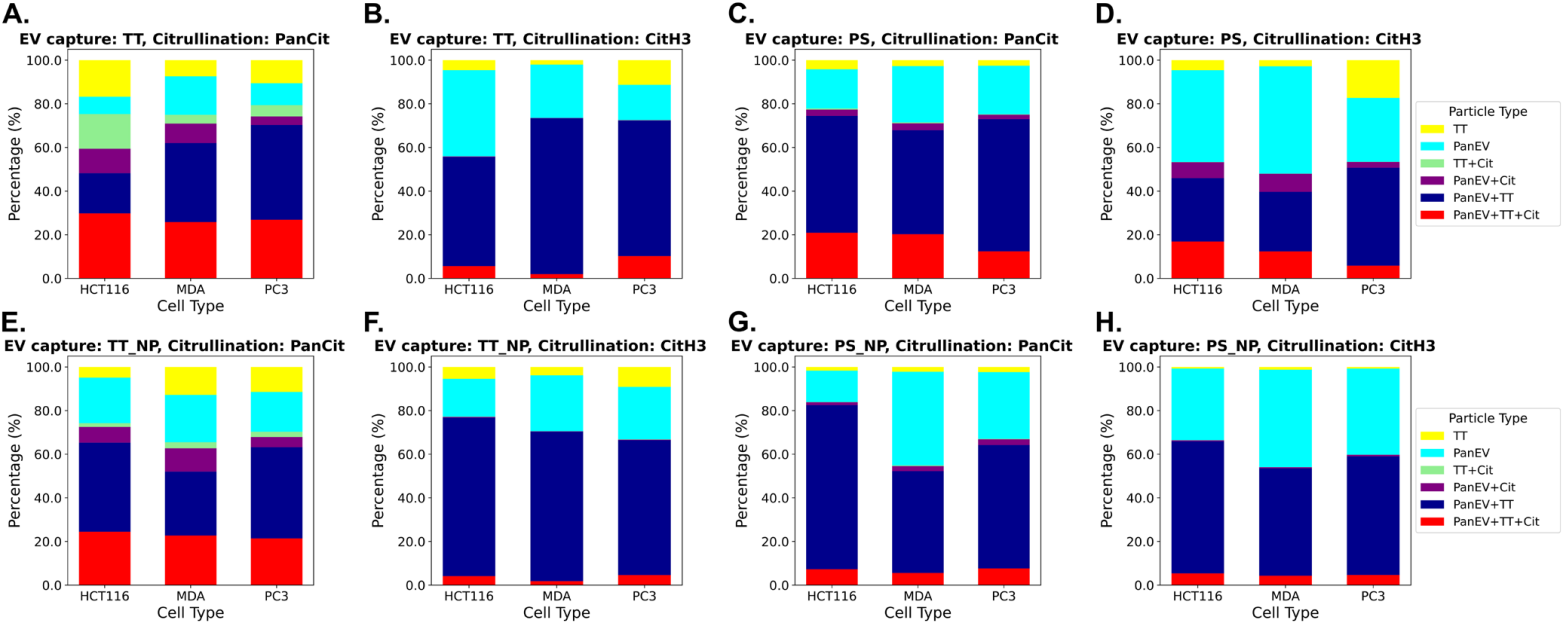
Distribution of citrullinated protein cargoes localised to EV subpopulations, comparing permeabilised (A–D) and non‐permeabilised (E–H) EVs, captured by TT or PS binding. The graphs show positive staining of EVs for pan‐citrullination (A,C,E,G) and CitH3 (B,D,F,H) respectively. The data is represented in stacked bar charts, showing the proportion of co‐stained EVs for pan‐EV, TT, and the citrullinated protein targets of interest (Cit), which were either PanCit or CitH3. EVs were either captured by TT (A,B,E,F) or by PS (C,D,G,H).

The proportion of positive citrullination signal (panCit or CitH3) is outlined in Figure 2 showing the distribution of positive signal for TT surface marker only, pan‐EV surface marker only, TT‐pan‐EV‐Citrullinated protein (cit—representative of either panCit or CitH3), or TT‐cit or PS‐cit positive EVs. Results are shown for permeabilised EVs (Figure 2A–D) for TT bound EVs (Figure 2A,B) and PS bound EVs (Figure 2C,D), respectively. Results are shown for non‐permeabilsed (NP) EVs (Figure 2E–H) for TT bound EVs (Figure 2E,F) and PS bound EVs (Figure 2G,H), respectively.

EVs were captured by either TT (Figure 2A,B,E,F) or PS (Figure 2C,D,G,H). The results are shown for all three cancer cell lines tested, including the standard HCT116 colon cancer–EVs provided with the EV profiler kit (ONI), compared with the PC3 EVs and MDA‐MB‐231 EVs.

As shown in Figure 2, positive detection of pan‐Cit and CitH3 was confirmed for both permeabilised (Figure 2A–D) and non‐permeabilised (Figure 2E–H) EVs, highlighting the presence of both intraluminal cargo and EV surface detection, with overall higher positivity for the permeabilised EVs. Permeabilised TT‐bound EVs co‐stained for pan‐EV and TT marker contained 25%–30% pan‐Cit staining, while those EVs positive for pan‐EV marker and pan‐Cit staining were 11%, and EVs co‐stained for TT and pan‐Cit staining were 4%–16% (Figure 2A).

When assessing the TT‐bound permeabilised EVs for CitH3 staining (Figure 2B), 2%–10% of CitH3 staining was observed for the TT and pan‐EV double‐positive EVs; but < 1% CitH3 detection for single‐labelled EVs only (Figure 2B).

For the PS‐bound permeabilised EVs (Figure 2C,D), panCit staining co‐localised 10%–20% to the double‐stained EVs (Figure 2C), with 2%–5% co‐localised to the pan‐EV labelled EVs, but no panCit staining was co‐localised to TT positive EVs only in this condition. Similarly, CitH3 staining (Figure 2D) was mainly colocalised to double‐stained EVs (5%–18%), and 3%–10% to pan‐EV positive EVs, but < 1% to TT positive EVs only (Figure 2D).

When assessing non‐permeabilised EVs, representative of cargo protein detection on the EV surface, the following was observed (Figure 2E,F,G,H): For TT captured EVs, 20%–25% EVs were positive for panCit staining co‐localised to TT and pan‐EV double‐staining. Approximately 5%–11% of the panCit stain colocalised to pan‐EV stain only, and 2%–3% to TT stain only, depending on cell line (Figure 2E). For the CitH3 labelled TT‐captured EVs, approximately 2%–5% were pan‐EV and TT double‐stained; no CitH3 detection was associated with single labelled (TT or pan‐EV only) EVs (Figure 2F).

For the PS‐bound non‐permeabilised EVs (EV surface/corona detection) (Figure 2G,H), panCit staining was detected in approximately 6%–8% of EVs double‐labelled for TT and pan‐EV marker, and approximately 2%–3% of pan‐Cit staining was localised to Pan‐EV labelled EVs (Figure 2G). When assessing the CitH3 labelling for these EVs, approximately 4% was co‐localised to EVs double‐labelled for TT and pan‐EV marker, and approximately 0.5% to pan‐EV labelled EVs only (Figure 2H).

It must be noted that some of the TT‐captured EVs showed single positive detection of the pan EV stain and not TT positivity. The EVs were captured using ONI's TetraTrio (TT) reagent, which immobilises vesicles via surface tetraspanins (CD9, CD63 and CD81). As these epitopes are involved in the capture interaction, subsequent fluorescent detection of tetraspanins bound to the same vesicles can possibly be reduced or absent due to epitope masking. Therefore, the absence of surface tetraspanin signal in a captured EV does not necessarily imply absence of the corresponding proteins but may reflect limitations inherent to antibody‐based capture on the same epitopes. As these inefficiencies were present in all EV populations studied, comparisons of expression between populations remain valid, even if absolute expression levels may be underestimated. Furthermore, the cut‐off for positive signal detection for each marker was set at 5 for the TT and pan‐EV markers, but at 2 for the protein cargos.

#### 3.2.2 Total Detection of Pan‐Citrullination Versus CitH3 EV Cargoes—Colocalised to EV Subtypes—Comparing Permeabilised Versus Non‐Permeabilised EVs

**FIGURE 3:**
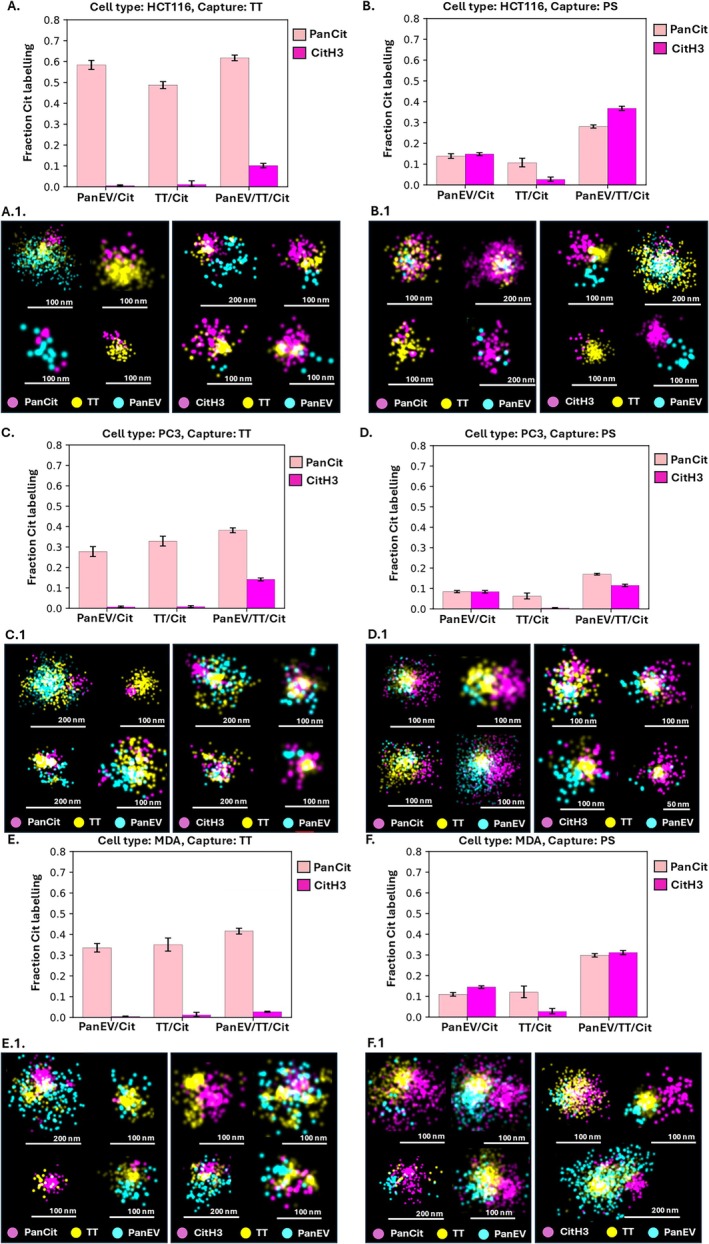
Assessment of citrullinated proteins in permeabilised EVs. Comparison of pan‐Cit and CitH3 staining colocalised to EV subtypes from HCT116, PC3 and MDA‐MB‐231 cells. Positive detection was assessed in EVs, captured by TT (A, C, E) or by PS capture (B, D, F); error bars represent standard deviation (SD). Scale bars for representative EV images (A.1–F.1) are indicated at 50, 100 and 200 nm, respectively.

**FIGURE 4:**
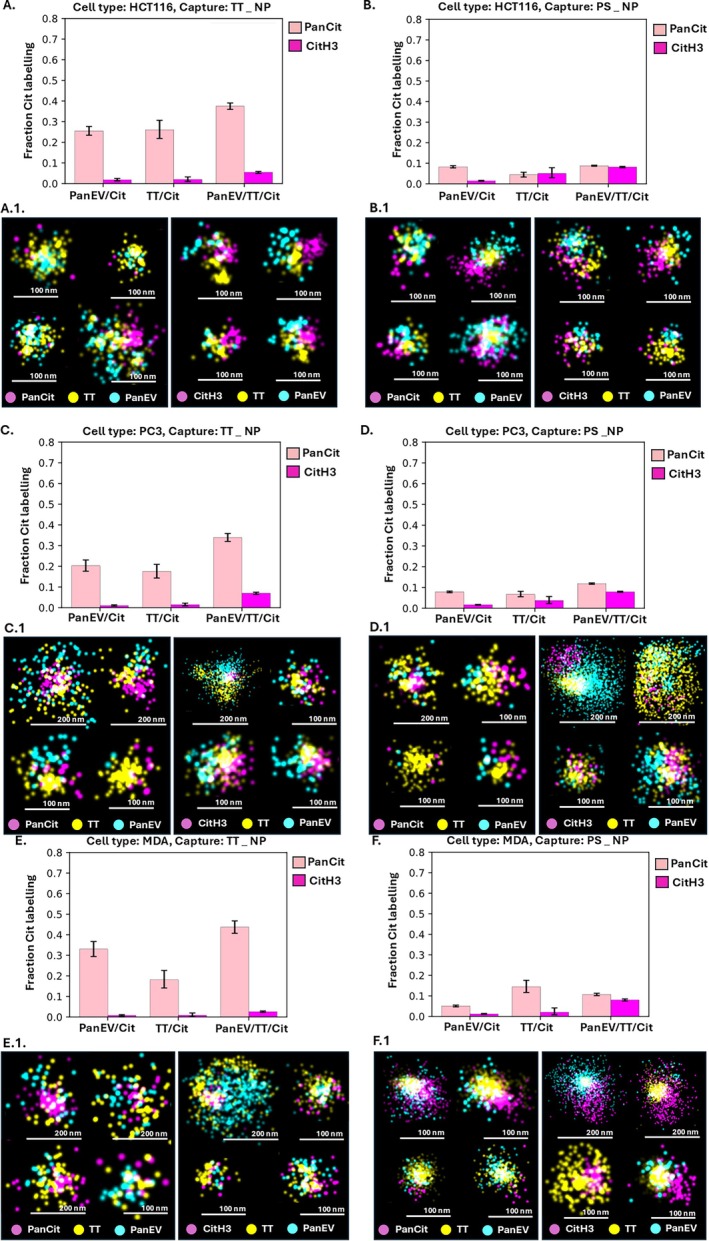
Assessment of citrullinated proteins in non‐permeabilised EVs. Comparison of panCit and CitH3 staining colocalised to EV subtypes from HCT116, PC3 and MDA‐MB‐231 cells. Positive detection was assessed in EVs, either captured by TT (A, C, E) or by PS capture (B, D, F); error bars represent standard deviation (SD). Scale bars for representative EV images (A.1–F.1) are indicated at 50, 100 and 200 nm, respectively.

Further assessment was carried out between the positive detection of panCit and CitH3 in permeabilised (Figure 3) versus non‐permeabilised EVs (Figure 4). This was colocalised to EV subtypes, based on surface marker detection (pan‐EV, TT positive) for EVs from the three cancer cell lines following either TT or PS capture of the EVs before staining.

Comparison of citrullinated protein detection is shown for the permeabilised EVs in Figure 3 as follows: HCT116 (Figure 3A,B), PC3 (Figure 3C,D), MDA‐MB‐231 (Figure 3E,F). EVs captured by TT are presented in Figure 3A,C,E, while those captured by PS are shown in Figure 3B,D,F. Representative dSTORM images of EVs with panCit or CitH3 positive signal are shown for each analysis and are correspondingly presented in Figure 3A.1–F.1. An example overview larger field‐of‐view dSTORM image is provided in Figure S1 to clarify that the observed co‐localisations of EV markers were a general phenomenon in the samples.

As presented in Figure 3, TT‐captured EVs from all three cell lines had significantly higher levels of pan‐EV staining (ranging from 30%–60% between cell lines), compared with CitH3 staining (ranging from 3%–15%, between cell lines, *p*_same_ < 0.001 Figure 3A,C,E). Co‐localisations for the permeabilised TT‐captured EVs (Figure 3A,C,E) furthermore showed that most panCit signal was colocalised to the pan‐EV‐TT double‐stained EVs (Figure 3A–E). When assessing the permeabilised PS‐captured EVs from all three cell lines (Figure 3B,D,F), panCit and CitH3 levels were similar in the pan‐EV labelled EVs, as well as in the pan‐EV‐TT double‐labelled EVs, while CitH3 positivity was considerably lower in the TT single labelled EVs, compared with the panCit signal (*p*_same_ < 0.001; Figure 3B,D,F).

Figure 4 shows the same analysis for non‐permeabilised EVs, which were compared for panCit and CitH3 staining, co‐localised to the EV subtypes. The results for non‐permeabilised EVs from all three cell lines are shown in Figure 4 as follows: HCT116 (Figure 4A,B), PC3 (Figure 4C,D), MDA‐MB‐231 (Figure 4E,F), also comparing EVs captured by TT (Figure 4A,C,E) or PS (Figure 4B,D,F), respectively. dSTORM images of EV types with panCit or CitH3 positive signal are shown for each set of graphs in Figure 4A.1–F.1.

As presented in Figure 4, non‐permeabilised TT‐captured EVs (Figure 4A,C,E) from all three cell lines had significantly higher positive signals for citrullinated proteins than the PS captured EVs (Figure 4B,D,F). For the TT captured EVs, positive pan‐Cit staining ranged from 18%–45% between cell lines, compared with citH3 staining which ranged from 1%–8%, between cell lines (*p*_same_ < 0.001; Figure 4A,C,E). The pan‐Cit positive signal was overall similar across all three EV co‐localisations for the TT‐captured EVs, although the highest levels were seen for the pan‐EV‐TT double‐stained EVs and the same pattern was observed for the CitH3 positive EVs (Figure 4A,C,E).

When assessing the non‐permeabilised PS‐captured EVs from all three cell lines (Figure 4B,D,F) there were fewer marked differences between panCit and CitH3 levels (all mean differences < 10%), with comparable or overall lower citH3 labelling in the pan‐EV labelled, compared with the pan‐EV and TT double labelled EVs (Figure 4B,D,F).

#### 3.2.3 Comparison of Permeabilised Versus Non‐Permeabilised EVs Highlights Differences in Citrullination Signal for Pan‐Citrullination and CitH3, Between Cancer Cell Types and EV Subtypes

**FIGURE 5:**
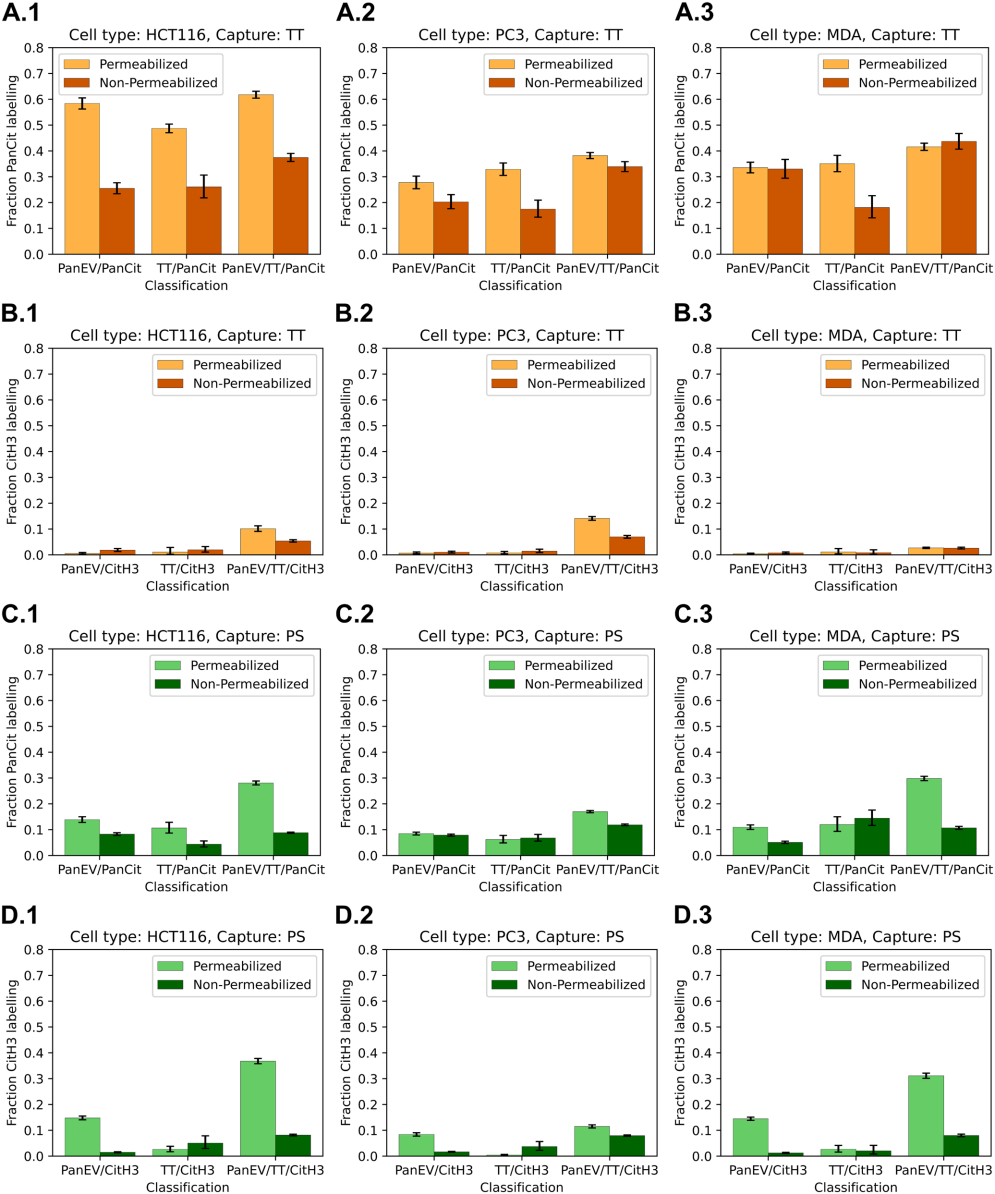
Proportional comparison of citrullinated protein detection (panCit or CitH3) in permeabilised versus non‐permeabilised EVs from HCT116, PC3 and MDA‐MB‐231 cells, showing TT and PS binding respectively. (A) PanCit labelling of TT captured EVs; (B) CitH3 labelling of TT captured EVs; (C) PanCit labelling of PS captured EVs; (D) CitH3 labelling of PS captured EVs. EVs from HCT116 cells are shown in A.1–D.1; from PC3 cells in A.2–D.2 and EVs from MDA‐MB‐231 cells in A.3–D.3. Error bars represent standard deviation (SD).

The EVs of all three cell lines were compared for panCit and CitH3 detection in permeabilised versus non‐permeabilised EVs, as presented in Figure 5. Analysis of TT captured EVs is shown for all three cell lines for panCit staining in Figure 5A, and for CitH3 staining in Figure 5B. This analysis showed some variability between cell lines, but overall, a higher proportion of panCit staining was found in the non‐permeabilised, versus the permeabilised EVs, in all EV sub‐populations (Figure 5A.1–A.3; *p*_same_ < 0.001). The EVs from the MDA‐MB‐231 cells (following TT capture) showed the least differences between permeabilised and non‐permeabilised conditions, with significant differences only in the TT‐labelled EVs (*p*_same_ < 0.001). For CitH3 staining, permeabilised EVs showed higher staining for the pan‐EV‐TT double‐labelled EVs for both HCT116 and PC3 EVs (Figure 5B.1,B.2; *p*_same_ < 0.001), while for MBA‐MD‐231 EVs no significant differences were observed between permeabilised versus non‐permeabilised EVs.

Analysis of the PS captured EVs is shown for all three cell lines for panCit staining in Figure 5C and for CitH3 staining in Figure 5D. Assessment of panCit staining showed higher levels in permeabilised versus non‐permeabilised EVs from HCT116 cells, for all three EV populations assessed (Figure 5C1, *p*_same_ < 0.001). PC3 EVs showed higher panCit levels in the double‐labelled pan‐EV‐TT EVs (*p*_same_ < 0.001), but no significant differences in the other two EV populations (Figure 5C2). In MDA‐MB‐231 EVs, panCit detection was significantly higher in the permeabilised EVs positive for pan‐EV marker and for pan‐EV + TT double‐labelled EVs (*p*_same_ < 0.001), while no significant differences were observed for permeabilised versus non‐permeabilised TT positive EVs (Figure 5C3). When assessing CitH3 staining in the PS‐captured EVs, significantly (*p*_same_ < 0.001) higher positive staining was observed in HCT116 permeabilised EVs labelled for panEV and panEV+TT, while no significant difference was observed for the TT‐stained EV population (Figure 5D.1). For the PC3 EVs (Figure 5D.2) and MDA‐MB‐231 EVs (Figure 5D.3) a similar pattern was observed, with significantly higher CitH3 labelling in the permeabilised EVs compared with the non‐permeabilised EVs labelled with pan‐EV and double‐labelled with pan‐EV + TT (*p*_same_ < 0.001).

### 3.3 Size Distribution Profiles of EVs Carrying Citrullinated Protein Cargoes—Assessing Permeabilised and Non‐Permeabilised Conditions, TT‐ and PS‐Capture, Respectively

**FIGURE 6:**
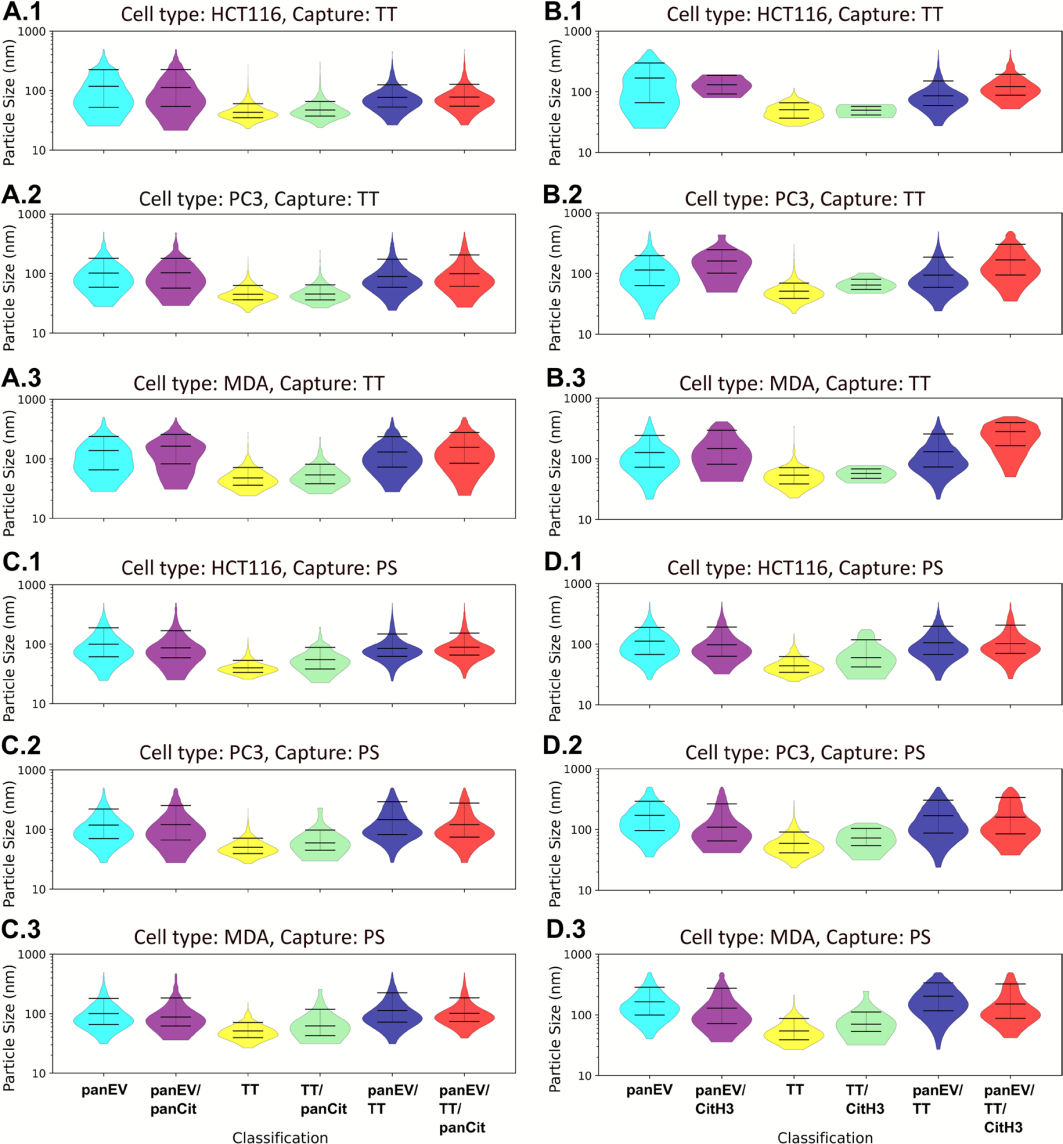
Violin plots showing size distribution profiles of EVs carrying citrullinated protein cargoes, assessing permeabilised EVs following TT‐ or PS‐capture. In each case subfigure 1 is HCT116, 2 is PC3 and 3 is MDA‐MB‐231. (A) PanCit staining for TT‐captured EVs. (B) CitH3 staining for TT‐captured EVs. (C) PanCit staining for PS‐captured EVs. (D) CitH3 staining for PS captured EVs. The mean is shown with the error bars representing standard deviation (SD).

**FIGURE 7:**
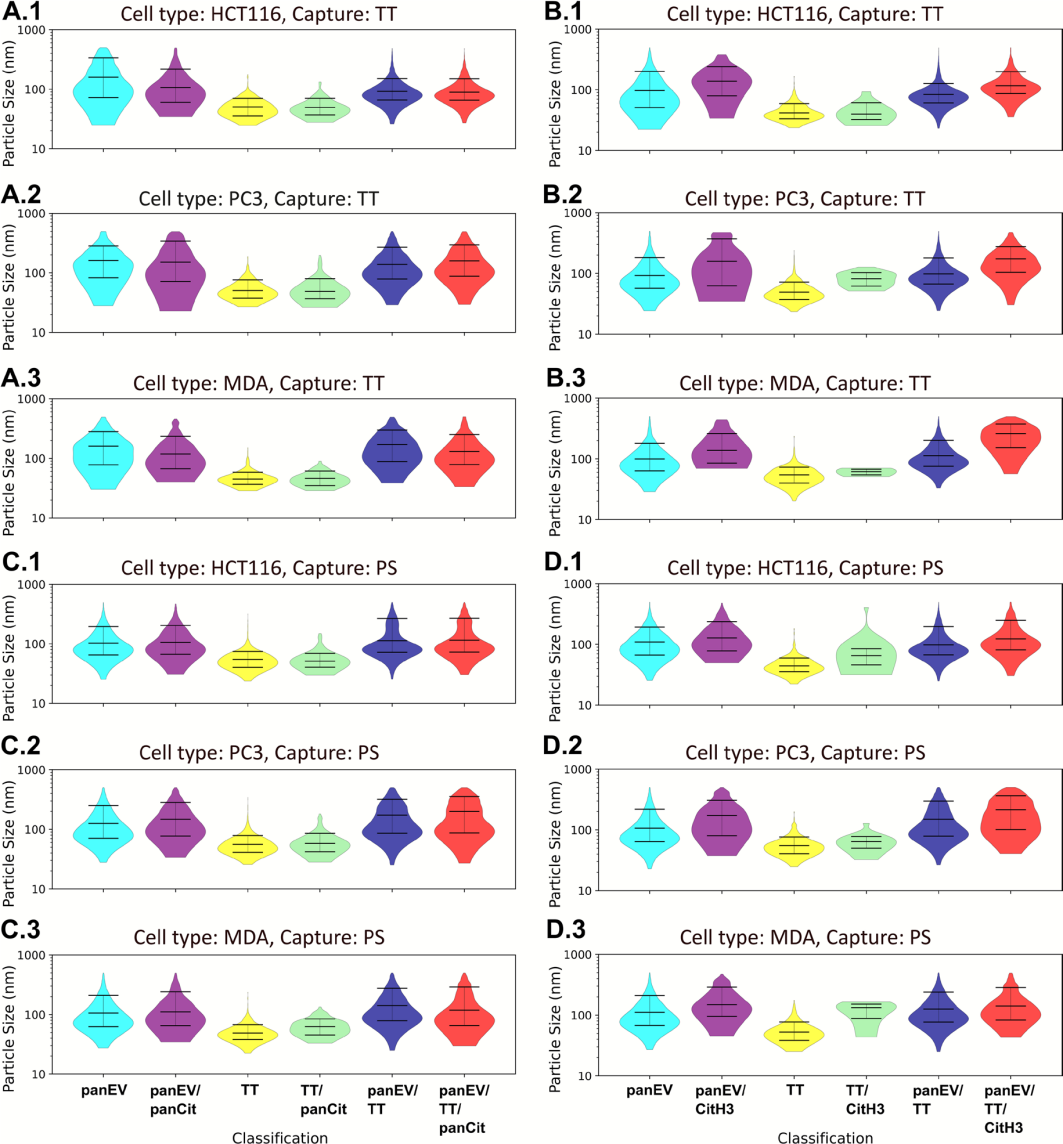
Violin plots showing size distribution profiles of non‐permeabilised EVs and associated citrullinated protein detection, TT and PS capture respectively. In each case subfigure 1 is HCT116, 2 is PC3 and 3 is MDA‐MB‐231. (A) PanCit staining for TT captured EVs. (B) CitH3 staining for TT captured EVs. (C) PanCit staining for PS captured EVs. (D) CitH3 staining for PS captured EVs. The mean is shown with the error bars representing standard deviation (SD).

The EVs labelled positively with the respective EV markers (TT or pan‐EV) and the citrullinated protein cargo of interest (panCit or CitH3) were further assessed for size distribution profiles within each labelled group, assessing permeabilised (Figure 6) and non‐permeabilised (Figure 7) EVs. Size distribution profiles correlated overall to the EV size distribution profiles observed by the NTA analysis shown in Figure 1.

#### 3.3.1 Assessment of EV Citrullinomes of Permeabilised EVs

The results presented in Figure 6 show the size distribution of permeabilised EVs, with respect to TT‐captured EVs (Figure 6A,B), labelled for panCit signal and EV subpopulations (Figure 6A) or labelled for CitH3 signal and EV subpopulations (Figure 6B), respectively. The PS‐captured permeabilised EVs were similarly assessed for size distribution profiles (Figure 6C,D), showing panCit staining (Figure 6C) and CitH3 staining (Figure 6D) colocalised to EV subpopulations, respectively.

Assessment of citrullinated cargoes to particle size distribution, representative of EV sizes, within each positively labelled group confirmed that TT positivity was associated with small EVs (sEVs ≤ 100 nm), while pan‐EV positivity, which labels all EVs, was associated with a wider size range of EVs, including sEVs (≤ 100 nm), medium EVs (mEVs 101–200 nm) and large EVs (lEVs > 200 nm). For the TT captured permeabilised EVs, pan‐EV/panCit positive EVs were at the size range of HCT116: median: 113 nm (CI_67%_: 52 to 226 nm), PC3: median: 101 nm (CI_67%_: 58 to 180 nm), and MDA‐MB‐231: median: 137 nm (CI_67%_: 65 to 238 nm), respectively. TT/panCit positive EVs were at the size range of HCT116: median: 77 nm (CI_67%_: 53 to 126 nm), PC3: median: 89 nm (CI_67%_: 58 to 174 nm), and MDA‐MB‐231: median: 130 nm (CI_67%_: 72 to 235 nm), respectively. The pan‐EV/TT/panCit positive EVs were at the size range of HCT: median: 78 nm (CI_67%_: 55 to 128 nm), PC3: median: 99 nm (CI_67%_: 61 to 205 nm), and MDA‐MB‐231: median: 156 nm (CI_67%_: 85 to 276 nm), respectively (Figure 6A.1–A.3).

A similar signal distribution was observed for the CitH3 co‐labelling to EV subtypes of the TT‐captured EVs. The pan‐EV/CitH3 positive EVs were at the size range of HCT116: median: 131 nm (CI_67%_: 92 to 186 nm), PC3: median: 161 nm (CI_67%_: 101 to 248 nm), and MDA‐MB‐231: median: 148 nm (CI_67%_: 82 to 295 nm), respectively. TT/CitH3 positive EVs were at the size range of HCT116: median: 50 nm (CI_67%_: 41 to 58 nm), PC3: median: 65 nm (CI_67%_: 55 to 81 nm), and MDA‐MB‐231: median: 58 nm (CI_67%_: 48 to 68 nm), respectively. The pan‐EV/TT/CitH3 positive EVs were at the size range of HCT116: median: 122 nm (CI_67%_: 88 to 193 nm), PC3: median: 167 nm (CI_67%_: 94 to 304 nm), and MDA‐MB‐231: median: 281 nm (CI_67%_: 166 to 397 nm), respectively (Figure 6B.1–B.3).

For the PS captured permeabilised EVs, pan‐EV/panCit positive EVs were at the size range of HCT116: median: 87 nm (CI_67%_: 59 to 168 nm), PC3: median: 121 nm (CI_67%_: 67 to 253 nm) MDA: median: 88 nm (CI_67%_: 62 to 185 nm) for HCT116, PC3 and MDA EVs respectively. TT/panCit positive EVs were at the size range of HCT: median: 55 nm (CI_67%_: 38 to 88 nm), PC3: median: 59 nm (CI_67%_: 45 to 98 nm) MDA: median: 62 nm (CI_67%_: 43 to 119 nm) for HCT116, PC3 and MDA EVs respectively. The pan‐EV/TT/panCit positive EVs were in the size range of HCT116: median: 89 nm (CI67%: 66 to 153 nm), PC3: median: 121 nm (CI_67%_: 75 to 280 nm), and MDA‐MB‐231: median: 102 nm (CI_67%_: 74 to 186 nm), respectively (Figure 6C.1–C.3).

For the PS captured permeabilised EVs, pan‐EV/CitH3 positive EVs were at the size range of HCT116: median: 98 nm (CI67%: 63 to 191 nm), PC3: median: 109 nm (CI_67%_: 65 to 266 nm), and MDA‐MB‐231: median: 129 nm (CI_67%_: 72 to 274 nm), respectively. The TT/CitH3 positive EVs were at the size range of HCT116: median: 60 nm (CI_67%_: 42 to 119 nm), PC3: median: 73 nm (CI67%: 54 to 104 nm), and MDA‐MB‐231: median: 70 nm (CI_67%_: 53 to 112 nm), respectively. The pan‐EV/TT/CitH3 positive EVs were at the size range of HCT116: median: 102 nm (CI_67%_: 71 to 207 nm), PC3: median: 160 nm (CI_67%_: 85 to 338 nm), and MDA‐MB‐231: median: 150 nm (CI_67%_: 88 to 322 nm), respectively (Figure 6D.1–D.3).

#### 3.3.2 Assessment of EV Citrullinomes of Non‐Permeabilised EVs

For non‐permeabilised EVs, the same analysis was performed for citrullinated signals (panCit and CitH3) on EVs from the three cell lines, relating to particle size distribution, and results are presented in Figure 7. Non‐permeabilised TT captured EVs are presented in Figure 7A,B, and non‐permeabilised PS captured EVs in Figure 7C,D.

For the TT captured non‐permeabilised EVs, pan‐EV/panCit positive EVs were in the size range of HCT116: median: 107 nm (CI_67%_: 60 to 218 nm), PC3: median: 152 nm (CI_67%_: 72 to 341 nm), and MDA‐MB‐231: median: 118 nm (CI_67%_: 67 to 234 nm), respectively. The TT/panCit positive EVs were in the size range of HCT116: median: 49 nm (CI_67%_: 37 to 70 nm), PC3: median: 49 nm (CI67%: 37 to 80 nm), and MDA‐MB‐231: median: 46 nm (CI_67%_: 35 to 61 nm), respectively. The pan‐EV/TT/panCit positive EVs were in the size range of HCT116: median: 89 nm (CI_67%_: 66 to 150 nm), PC3: median: 159 nm (CI_67%_: 88 to 295 nm), and MDA‐MB‐231: median: 130 nm (CI_67%_: 78 to 251 nm), respectively (Figure 7A.1–A.3).

A similar distribution was seen for the CitH3 co‐labelling of TT‐bound EVs to EV subtypes. The pan‐EV/CitH3 positive EVs were at the size range of HCT116: median: 138 nm (CI_67%_: 79 to 241 nm), PC3: median: 159 nm (CI_67%_: 63 to 370 nm), and MDA‐MB‐231: median: 138 nm (CI_67%_: 85 to 260 nm), respectively. The TT/CitH3 positive EVs were at the size range of HCT116: median: 40 nm (CI_67%_: 32 to 61 nm), PC3: median: 82 nm (CI_67%_: 62 to 103 nm), and MDA: median: 61 nm (CI_67%_: 54 to 67 nm), respectively. The pan‐EV/TT/CitH3 positive EVs were at the size range of HCT116: median: 117 nm (CI_67%_: 87 to 198 nm), PC3: median: 174 nm (CI_67%_: 104 to 277 nm), and MDA‐MB‐231: median: 260 nm (CI_67%_: 152 to 375 nm), respectively (Figure 7B.1–B.3).

For the PS captured non‐permeabilised EVs, pan‐EV/panCit positive EVs were in the size range of HCT116: median: 106 nm (CI67%: 67 to 204 nm), PC3: median: 148 nm (CI67%: 77 to 284 nm), and MDA‐MB‐231: median: 111 nm (CI67%: 65 to 241 nm), respectively. The TT/panCit positive EVs were in the size range of HCT116: median: 51 nm (CI67%: 40 to 69 nm), PC3: median: 58 nm (CI67%: 42 to 86 nm), and MDA‐MB‐231: median: 63 nm (CI_67%_: 45 to 84 nm), respectively. The pan‐EV/TT/panCit positive EVs were in the size range of HCT116: median: 115 nm (CI_67%_: 73 to 269 nm), PC3: median: 199 nm (CI_67%_: 87 to 355 nm), and MDA‐MB‐231: median: 119 nm (CI_67%_: 65 to 290 nm), respectively (Figure 7C.1–C.3).

For the PS captured non‐permeabilised EVs, pan‐EV/CitH3 positive EVs were at the size range of HCT116: median: 128 nm (CI_67%_: 78 to 238 nm), PC3: median: 173 nm (CI_67%_: 80 to 309 nm), and MDA‐MB‐231: median: 149 nm (CI_67%_: 96 to 289 nm), respectively. The TT/CitH3 positive EVs were at the size range of HCT116: median: 65 nm (CI_67%_: 46 to 85 nm), PC3: median: 64 nm (CI67%: 50 to 78 nm), and MDA‐MB‐231: median: 132 nm (CI_67%_: 89 to 152 nm), respectively. The pan‐EV/TT/CitH3 positive EVs were at the size range of HCT116: median: 123 nm (CI_67%_: 81 to 250 nm), PC3: median: 215 nm (CI67%: 101 to 365 nm), and MDA‐MB‐231: median: 141 nm (CI_67%_: 83 to 284 nm), respectively (Figure 7D.1–D.3).

## 4 Discussion

This is the first study to co‐localise post‐translationally citrullinated/deiminated protein cargoes to EVs and EV subtypes, using EVs from three cancer cell lines—HCT116 (colorectal carcinoma), PC3 (prostate cancer) and MDA‐MD‐231(breast adenocarcinoma) as examples. We assessed positive signals for panCit proteins and CitH3 in EVs at the nanometre scale, by single molecular detection using dSTORM, in both permeabilised and non‐permeabilised EVs. This allows comparison of intraluminal protein EV cargoes to proteins carried on the EV surface (EV corona). Overall, stronger signals for citrullinated proteins were detected in the permeabilised compared with the non‐permeabilised EVs, indicative of higher levels of intraluminal export. It may be postulated that detergent permeabilisation could alter vesicle integrity by partial solubilisation of membrane lipids or osmotic imbalance. Here, we used a mild 0.1% Triton X‐100 treatment (5 min) following the manufacturer's (ONI) EV profiling protocol, which has previously been shown to maintain vesicle morphology by dSTORM [49]. Comparative Pan‐EV size distributions of permeabilised versus non‐permeabilised preparations furthermore showed median differences < 5%, which is far smaller than the expected localisation‐based measurement error. While minor structural changes cannot be ruled out, they are unlikely to influence the comparative trends reported here.

PanCit protein detection was significantly higher in EVs from all three cancer cell lines, compared with CitH3 detection, confirming that CitH3 forms part of the total citrullinome cargo. Our findings expand the landscape of EV‐associated post‐translational modifications and establish a methodological framework for citrullination profiling in EVs, with translational potential for EV‐based liquid biopsy tools in cancer and inflammatory disease.

The use of EVs and various specific EV cargo contents, as liquid biopsy tools, has been highlighted for many cancers, including colorectal cancer [50, 51, 52, 53], prostate cancer [54, 55, 56, 57, 58] and breast cancer [59, 60, 61, 62]. While many studies have assessed changes in protein EV cargoes, studies on post‐translational proteins, including citrullination signatures are still scarce. Previous work from our group has highlighted roles for PADs and post‐translational citrullination in several cancers, including breast, prostate, brain and pancreatic cancer [24, 25, 27, 30]. Furthermore, the role for CitH3 in cancers has received considerable attention, including as an indicative biomarker in cancer patients [18, 19, 20, 21, 22, 23]. To date no detailed evaluation has been carried out on EV‐mediated transport of CitH3, including in EV subtypes, while proteomic data analyses from our group on EVs from various body fluids have identified CitH3 as part of total EV cargoes. As in this current study CitH3 was mostly localised to the permeabilised EVs; this indicates that previous reports of CitH3 as a circulatory cancer biomarker [18, 19, 20] may be strongly associated with intraluminal EV‐mediated export, informing refinement of current liquid biopsy approaches.

Using single‐molecule detection with the EV profiler kit and ONI nanoimager system, analyzing post‐translational citrullinated proteins in EVs at the nanometre scale, allows for the first time a detailed analysis of EV citrullinomes and co‐localisation to EV subtypes, based on tetraspanin and pan‐EV markers, using three standard cell lines commonly used for colon (HCT116), prostate (PC3) and breast cancer (MDA‐MB‐231) research. This verifies the sensitivity of the methodological approaches developed here to distinguish EV cargo differences between cell lines, in this case representative of cancer types. Importantly, the presented EV citrullinome analysis for in vitro cancer cell models may offer a translatable tool for other PAD/citrullination associated pathologies, including autoimmune, inflammatory and neurodegenerative conditions [12, 13, 14, 15, 16, 17, 36, 42].

Profiling of post‐translational modifications in EVs is a growing area of research, with recent studies assessing EV profiles for glycosylation [63, 64, 65, 66] and phosphorylation [67, 68, 69]. Furthermore, roles for different post‐translational modifications (including acetylation, glycosylation, methylation, phosphorylation and SUMOylation) in the packaging of EV cargoes, such as non‐coding RNAs, have also been reported [70, 71, 72, 73]. The current EV citrullinome analysis therefore further expands our current understanding of the complex landscape of post‐translational modifications in modulating disease mechanisms via EV‐mediated export in cell–cell communication.

In this study, the robust analysis of EV datasets generated by CODI, in conjunction with the EV Profiler Kit, was enhanced through the application of a Bayesian framework using beta‐distributed posteriors for binomial outcomes, alongside a two‐sample Kolmogorov–Smirnov (KS) test with bootstrap‐estimated median differences for population size distribution analysis. Together, these complementary techniques provide a rigorous basis for validation of single molecular detection data sets from dSTORM analysis of EV cargoes and offer an additional tool for extracting robust, quantitative insights from current standard EV Profiler Kit outputs. This approach may be particularly valuable for research and clinical applications, enabling more reliable assessment of EV‐linked biomarkers across a range of pathological conditions.

## 5 Conclusion

In conclusion, this is the first study to identify citrullinated proteins co‐localised to EV subtypes, using super resolution microscopy. Our findings expand current understanding of the complex epigenomic roles of post‐translational modifications in EV mediated cellular communication. The Bayesian framework presented here provides a new tool for modelling EV cargoes to EV subtypes, aiding the development of refined EV based liquid biopsy tools. While our present work was conducted in vitro using three established cancer cell lines, future validation should focus on extending these approaches to patients' biofluids, refining antibody tools for citrullination detection, and benchmarking against orthogonal methods such as LC–MS/MS, as well as comparing dSTORM‐based EV subtyping results with nano‐flow cytometry. These steps will help translating our findings into clinically robust EV‐based liquid biopsy platforms.

## Author Contributions

**S.R.N.:** formal analysis, data curation, investigation, methodology, resources, validation, visualization, writing – review and editing. **B.M.D:** formal analysis, data curation, investigation, methodology, software, resources, validation, visualization, writing – review and editing. **P.U.‐O.:** methodology, resources, writing – review and editing. **D.J.R:** formal analysis, methodology, validation, writing – review and editing. **M.H:** resources, writing – review and editing. **I.K:** methodology, resources, visualization. **J.M.I:** investigation, methodology, resources, writing – review and editing. **S.L:** conceptualization, data curation, formal analysis, funding acquisition, investigation, methodology, project administration, resources, supervision, validation, visualization, writing – original draft, writing – review and editing.

## Funding

This research was supported by funding from UKRI through access to STFC's Central Laser Facility's Octopus Facility (LSF 25130012).

## Conflicts of Interest

The authors declare no conflicts of interest.

## Supporting information


**Figure S1:** An example overview of a larger field‐of‐view dSTORM image in (A) and a closer‐up view of individual EVs in (B). The panels show PS captured permeabilised EVs from MDA‐MB‐231 cells labelled for PanCit (magenta), PanEV (blue) and TT (yellow) markers. Scale bars are indicated at 20 μm and 400 nm, respectively.

## Data Availability

The data that support the findings of this study are available from the corresponding author upon reasonable request.
