# Supplementary material for: Profiling Protein Citrullination in Extracellular Vesicles by Single‐Molecule Detection Using Direct Stochastic Optical Reconstruction Microscopy
Source: J Biophotonics. 2025 Nov 27;19(3):e202500483. doi: 10.1002/jbio.202500483 (PMC12976978; doi:10.1002/jbio.202500483)
Supplement: Supplementary file 1 — Figure S1: An example overview of a larger field‐of‐view dSTORM image in (A) and a closer‐up view of individual EVs in (B). The panels show PS captured permeabilised EVs from MDA‐MB‐231 cells labelled for PanCit (magenta), PanEV (blue) and TT (yellow) markers. Scale bars are indicated at 20 μm and 400 nm, respectively. [file JBIO-19-e202500483-s001.docx]

Supporting Information

Profiling Protein Citrullination in Extracellular Vesicles by Single-molecule Detection using direct Stochastic Optical Reconstruction Microscopy

Sarah R. Needham^1^, Benjamin M. Davis^1^, Pinar Uysal-Onganer^2^, Daniel J. Rolfe^1^, Mariya Hristova^3^, Igor Kraev^4^, Jameel M. Inal^5^, Sigrun Lange^6^*

*Correspondence:

Sigrun Lange, Pathobiology and Extracellular Vesicles Research Group, School of Life Sciences, University of Westminster, London W1W 6UW, UK

Email: s.lange@westminster.ac.uk


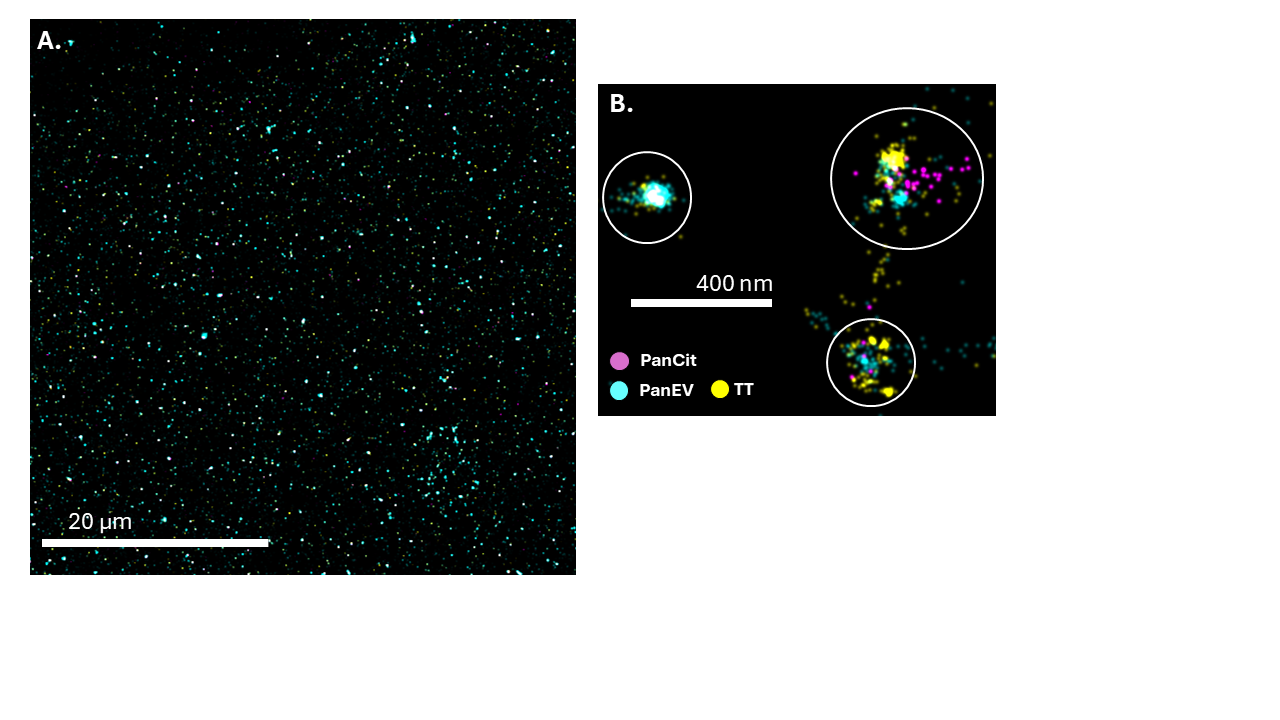


**Figure S1.** An example overview of a larger field-of-view dSTORM image in (A) and a closer-up view of individual EVs in (B). The panels show PS captured permeabilised EVs from MDA-MB-231 cells labelled for PanCit (magenta), PanEV (blue) and TT (yellow) markers. Scale bars are indicated at 20 µm and 400 nm, respectively.
